# Clinical Characteristics and Risk Factors of Geographic Tongue: A Retrospective Analysis of 100 Polish Patients

**DOI:** 10.3390/healthcare13111299

**Published:** 2025-05-29

**Authors:** Zuzanna Ślebioda, Julia Drożdżyńska, Aleksandra Karpińska, Aleksandra Krzyżaniak, Marianna Kasperczak, Natalia Tomoń, Paulina Wiśniewska, Marzena Liliana Wyganowska

**Affiliations:** 1 Department of Periodontology and Oral Mucosa Diseases, Poznan University of Medical Sciences, 60-812 Poznań, Poland; klchirstomiper@ump.edu.pl; 2 Medical Faculty, Poznan University of Medical Sciences, 60-812 Poznań, Poland; 86146@student.ump.edu.pl (J.D.); 82995@student.ump.edu.pl (A.K.); 86526@student.ump.edu.pl (A.K.); 80941@student.ump.edu.pl (M.K.); 85720@student.ump.edu.pl (N.T.); 77241@student.ump.edu.pl (P.W.)

**Keywords:** geographic tongue, oral mucosa, oral pathology

## Abstract

**Background/Objectives:** We aimed to evaluate the clinical course, demographic characteristics, and risk factors in Polish patients with geographic tongue (GT). **Methods:** The analysis was based on medical records of 100 patients with GT referred to the outpatient clinic of Poznań University of Medical Sciences (PUMS) from 2013 to 2023. Data regarding age, gender, medical history, habits, subjective complaints, clinical features, localization, histology, and treatment were considered. **Results:** Patients with GT constituted 11.3% of 887 individuals admitted to the outpatient clinic in the analyzed period. The female-to-male ratio was 52:48. The average age at diagnosis was 51.6 years. Thirteen patients reported smoking, and 2.0% admitted to consuming alcohol excessively. Subjective complaints were reported by 85.0% of patients and mainly included a burning sensation (57.0%), pain (39.0%), xerostomia (22.0%), bleeding (4.0%), and taste disturbance (3.0%), while 15.0% of GT patients were asymptomatic. Comorbidities were found in 76.0% of subjects with GT, and included cardiovascular disorders (37.0%), gastrointestinal and thyroid gland diseases (24.0% and 18.0%), and type II diabetes (15.0%). Psoriasis was observed in one case only. **Conclusions:** The frequency of GT in a Polish cohort of patients was high and comparable in both genders. The majority of participants reported subjective complaints, and most of the patients were non-smokers. Comorbidities were found in 76.0% of subjects with GT and mainly included cardiovascular and gastrointestinal diseases. GT was often accompanied by other oral conditions, like candidiasis, recurrent aphthous stomatitis, and lichen planus. GT screening should include cardiovascular and gastrointestinal evaluation.

## 1. Introduction

First described by Reiter in 1831, geographic tongue (GT), also known as benign migratory glossitis or *erythema migrans*, is a chronic relapsing inflammatory condition of the oral cavity. Characterized by irregular, erythematous, depapillated patches with serpiginous white borders, GT shows a map-like appearance and presents dynamic migratory behavior with remissions and exacerbations [1,2,3,4]. More diffuse lesions may appear less often, affecting oral mucosa sites other than the lingual one. This condition is then diagnosed as geographic stomatitis (GS), migratory stomatitis, or ectopic geographic tongue, first recognized in 1955 [5]. Both GT and GS are considered inflammatory conditions with psoriasiform patterns of lesions [1]. A classification of GT and GS lesions was first proposed by Hume in 1975, and recently modified by Netto et al., who enriched it with two new classes. A comparison of these two categorizations is shown in Table 1 [1,6].

Geographic tongue is often considered asymptomatic; however, some patients report subjective complaints. These symptoms are usually mild but can sometimes impact quality of life [2,7,8,9,10,11].

The prevalence of geographic tongue is 1–2.5% of the general population, reaching up to 14.3% in children [9,12,13]. The highest incidence of GT, at about 39.4%, was reported in the age group of 20–29 years, with a slight female predilection [14,15]. The patient population varies across countries, which may be explained by the type of food consumed, gastrointestinal and autoimmune diseases, the distribution of genetic variation, oral health status, and hygiene [16,17,18].

The precise etiology and pathogenesis of GT remain elusive. Several factors have been proposed, including genetic predisposition, stress, hormonal influences, and associations with systemic conditions [2,7]. Studies have demonstrated a higher prevalence of GT in individuals with psoriasis, suggesting a possible shared pathogenic mechanism, histological similarity, and a common genetic marker—HLA-Cw6 [7,16,19,20,21,22,23]. The role of HLA-DR5, HLA-DRW6 was also postulated in [24]. An increased incidence of tissue type HLA-B15 was found in GT patients with atopy in [25]. The genetic background of GT could include single-nucleotide polymorphisms in specific genes [22], autosomal dominant IL36RN gene mutations with incomplete penetrance [26], and a recently reported novel mutation in the ATP5MK gene [27]. An antigen’s presence promotes molecular mimicry and accelerates T lymphocyte proliferation [28]. Some studies have also shown that elevated levels of pro-inflammatory cytokines such as tumor necrosis factor-alpha (TNF-α) and interleukins 6, 8, 17, and 23 (IL-6, -8, -17, -23) trigger these conditions [22,29,30]. GT has also been associated with atopy, allergy, anemia, gastrointestinal disturbances, diabetes mellitus, celiac disease, and occasionally drug use [8,13,31,32,33,34]. Recent reports indicate the association of GT with antiangiogenic agents, which may contribute to the increased incidence of GT among oncology patients [35,36,37,38]. Psychological factors may have a significant impact on the prevalence of GT; lesions occur more frequently in mentally ill patients. Stress is regarded as a potentially modifiable risk factor. Reports suggest symptom reduction through stress management [13]. Stress may be associated with syndromes like Reiter’s, Down, Aarskog, Fetal hydantoin, and Robinow’s syndromes [13].

Microbial factors, mainly the presence of fungi, have also been associated with GT. It remains controversial whether the microbial shift is a direct consequence of GT, a causative factor, or a modifier of disease progression [12]. *Candida* has been commonly found in GT lesions. The thin, red patches of geographic tongue can become more susceptible to *Candida* infection, making the tongue more prone to thrush-like symptoms [9,24,39]. However, in general, GT is not considered an infectious condition.

The diagnosis is mostly clinically made, given the peculiar appearance of the lesions and their characteristic, temporary nature [40,41]. A histopathologic evaluation of the white area of the lesion reveals subepithelial infiltrates with a predominance of neutrophils and the formation of microabscesses or pustules with flaking necrotic cells. The erythematous area of GT shows mononuclear subepithelial infiltrate, suprapapillary hypertrophy, and vascular ectasia, accompanied by the loss of filiform papillae [2,7,42]. Differential geographic tongue diagnoses include erythroplakia, lichen planus, candidiasis, contact stomatitis, leukoplakia, trauma, and recurrent aphthous stomatitis [1,3,13].

Management is primarily conservative, focusing on alleviating symptoms with some dietary modifications [40,41]. Avoiding irritants such as dentures, braces, and acidic and spicy foods is also recommended [43].

While geographic tongue itself does not appear to directly increase the risk of cancer, any persistent, changing, or unusual lesions on the tongue should be evaluated to rule out malignancy [7,44].

The epidemiologic data from our region regarding GT’s characteristics and correlations with possible causative factors are scarce. The aim of our study was to retrospectively analyze the clinical manifestations of GT, its demographic characteristics, and the role of systemic and environmental risk factors in a group of 100 Polish patients with GT from the Poznan region.

## 2. Materials and Methods

We performed a retrospective chart review of patients treated at the Department of Dental Surgery, Periodontology and Oral Mucosa Diseases, Poznań University of Medical Sciences (PUMS), from 1 January 2013 to 31 December 2023. The study group comprised 100 patients diagnosed with GT referred to the student outpatient clinic, constituting 11.3% of the 886 analyzed records. Data regarding age, gender, chief complaints, localization, histology, and treatment were registered. Information on medical history, drugs, smoking, and alcohol consumption was also collected. The diagnosis of GT was based on clinical features and history, with a biopsy performed in atypical or refractory cases. Data collected from original medical records were introduced to and organized in MSExcell^®^ spreadsheets by two authors and presented descriptively. The validation of the process comprised verification of the Excel-introduced data by comparison with the original records, checking for missing information, verifying codes for diagnoses and treatments, and ensuring consistent data collection across sources. It was performed by the other two authors. A difference test between two proportions was used where appropriate, with *p* values lower than 0.05 considered significant. We used Dell Statistica (data analysis software system), version 13 (Dell Inc., 2016; Palo Alto, CA, USA).

## 3. Results

We reviewed the medical records of 100 patients diagnosed with geographic tongue. The gender distribution was almost equal; 52.0% of GT patients were females, and 48.0% were males (*p* = 0.5716). The mean age of the participants was 51.6, and it was higher in females than in males, with a significant difference (*p* = 0.0126).

The essential demographic characteristics of the study group are presented in Table 2 and Table 3.

Significantly more study participants reported urban residence (81.0%) and were non-smokers (87.0%).

Symptoms were reported by 85.0% of all study participants, and some of them described more than one complaint at a time. Complaints were reported significantly more often in patients aged over 40 years. In this age group, xerostomia and a burning sensation were more frequent than in younger individuals. Burning was also more often reported by females than by males. The most commonly reported symptoms included burning, pain, and xerostomia. Other occasionally reported subjective symptoms were bleeding from the oral mucosa, taste disturbances, paresthesia, and excessive saliva production.

**Figure 1:**
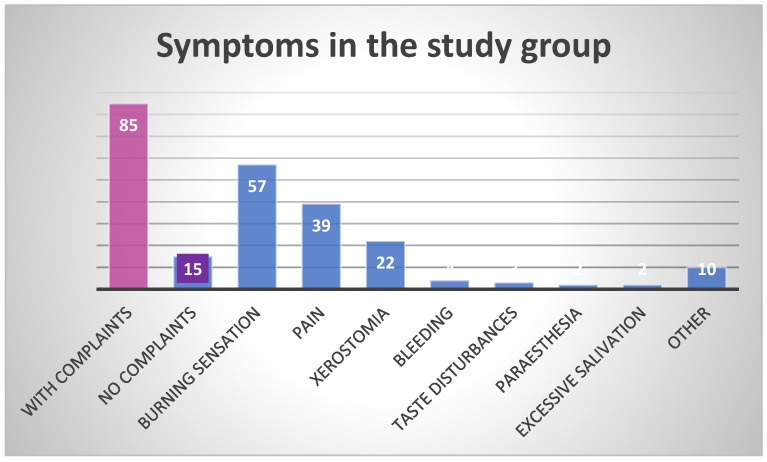
Frequency of symptoms in the study group.

Figure 1 shows the number and type of symptoms in the study population.

Table 4 illustrates the stratification of symptoms by gender and age group.

**Figure 2:**
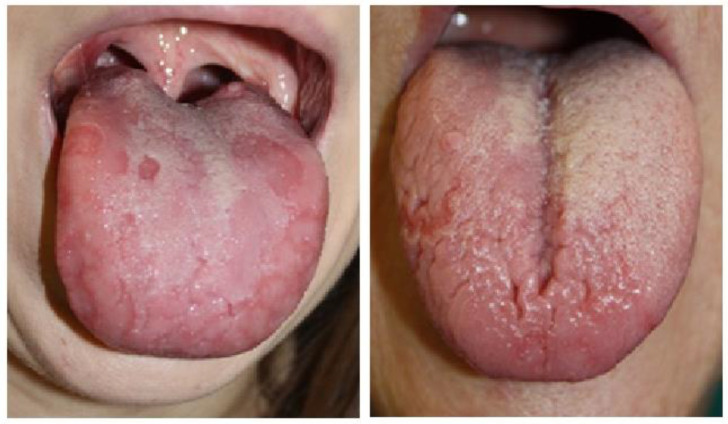
Variants of geographic tongue in patients from the study group: diffuse, irregularly shaped red patches with the loss of filiform papillae surrounded by circinate borders of the overgrown epithelium, accompanied by deep fissuring of the dorsal tongue surface, and indicating an active, severe phase of inflammation.

**Figure 3:**
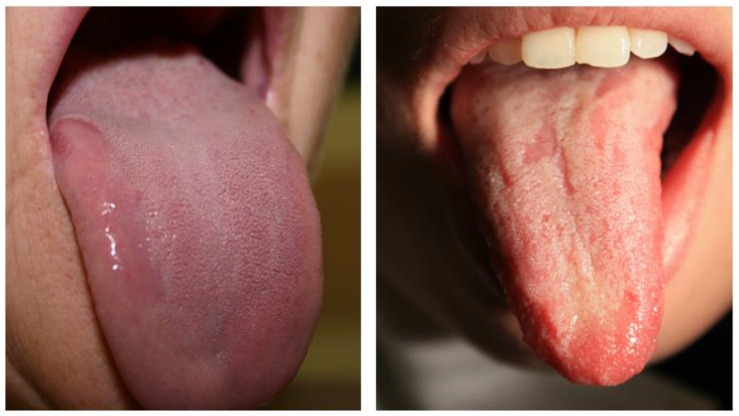
Variants of geographic tongue in patients from the study group: mild type of GT showing as single or multiple depapilated red patches on the dorsal tongue surface.

Figure 2 and Figure 3 depict various clinical presentations of GT observed in patients from the study group.

Histopathologic examination was performed on two patients from the study group, one female and one male. The results are presented in Table 5.

Table 6 shows the prevalence of oral mucosal lesions other than GT found in our study group. We included both strictly pathologic lesions and findings classified as abnormalities not qualifying to be treated.

The most common oral mucosal lesion that accompanied GT was candidiasis. Other frequently found conditions included recurrent aphthous stomatitis and lichen planus. Fissured tongue (FT) and *linea alba*, which were found to occur in a wide range of normal oral mucosa, were observed in six persons each. One patient was diagnosed with a tumor of the oral cavity, located on the palate and in the vestibule. He was, however, referred to the maxillofacial surgery department, and further procedures were conducted there with no follow-up in our facility.

Table 7 depicts systemic diseases revealed in the study group. At least one systemic condition was reported by 76.0% of patients with GT; of those, the most frequently reported were cardiovascular disorders (37.0%), gastrointestinal and thyroid gland diseases (24% and 18%), and diabetes type II (15.0%). Systemic diseases were revealed more often in patients over 40 years old. In this age group, cardiovascular and gastrointestinal disorders were significantly more frequent than in younger participants. Thyroid gland diseases were found more often in females than in males. Psoriasis was observed in one case only. The most common cardiovascular disorder was hypertension (32.0%). Frequently observed gastrointestinal diseases include gastroesophageal reflux (14.0%), celiac disease (5.0%), and irritable bowel disease (3.0%).

Treatment of GT was performed in 76 patients (76.0%), and some received more than one drug at a time. The most common therapeutic strategy included a herbal coating mouth-rinse, composed of flax seed, chamomile, mallow, and marshmallow, mixed in equal proportions (40 patients). Topical analgesics (lidocaine, benzydamine) were administered in 24 cases. Topical steroids were administered in 15 cases and included dexamethasone (13) and hydrocortisone (2). None of the patients qualified for systemic treatment with steroids. Non-steroidal anti-inflammatory topical agents were used in 13 cases. Eleven patients with GT received topical antifungals. Topical vitamin A or A+E was applied in nine cases, coating/covering agents in seven cases, moistening topical agents in six cases, and low-level laser therapy in four patients. Chlorhexidine 0.12% mouth rinse was used in three cases. Vitamin B12 (systemically) was prescribed in one case, as was clonazepam.

## 4. Discussion

Although GT is considered a benign, harmless, and transient oral disease, the lack of a precise definition of the etiology, together with a rather unique clinical presentation, may produce anxiety in several patients, some of them developing carcinophobia. Thus, stress, which has been regarded as an important etiologic factor in GT, may also represent an outcome of this condition [2].

The frequency of GT in our study was 11.3%, though the total study population comprised only the patients referred to the PUMS outpatient dental clinic. We enrolled all of the individuals diagnosed with GT based on the chart analyses conducted when they attended PUMS from 2013 to 2023. The frequency in this group may not strictly correspond to that in the general Polish community. The prevalence of geographic tongue is around 1–2.5% of the worldwide population [9,12,13], and according to some reports, it is reaching 4.8% [2,7]. As postulated by Hume, because of its nature, GT may be underdiagnosed if the result of only one examination is considered. The author suggests that it is necessary to re-examine patients suspected to have GT at certain time intervals to establish a final diagnosis [6]. This also applies to several other oral mucosa diseases like recurrent aphthous stomatitis and lichen planus. The patients included in this study did not attend regular follow-ups. Our analysis’s lack of longitudinal data limits insight into GT’s chronicity and symptom progression in the study population. As shown in Jainkittivong et al. and Derwazeh et al.’s studies, this condition is more frequently reported in adults than in children, with the highest incidence of about 39.4% reported in the age group of 20–29 years, and with a slight female predilection [14,15]. However, a contrary opinion was stated in Picciani et al.’s review and Sing et al.’s study, which described GT as occurring more often in children, with its frequency reducing with age [7,45]. The mean age of our patients was higher and reached 51.6 years [range: from 16 to 101]. The sex distribution among the GT patients in our study was almost equal. This aligns with the historic data presented by Hume [6]. Contrary to this, in Singh et al.’s study, there was a significant male predilection for GT among patients with psoriasis [45]. In contrast, a slight female predilection for GT was reported by Jainkittivong et al. and Derwazeh et al. [14,15].

Subjective complaints were reported by 85% of our study’s GT patients. The most commonly observed symptoms included burning and pain. According to several authors, GT is, in most cases, asymptomatic [2,7]. However, the frequency of symptoms reported in this study shows that GT may significantly influence life quality, forcing patients to seek medical help to reduce discomfort. In a comprehensive review by González-Álvarez et al., a burning sensation appeared in 9.2% to 47% of patients with GT. In their study, dysgeusia did not appear statistically more significant in GT compared to controls, potentially because lingual atrophy mainly affects the filiform papillae that are not involved in taste and preserves the fungiform papillae that contain the taste buds [46].

Nevertheless, the chronic course of the condition very often raises anxiety in patients, sometimes leading to carcinophobia. The therapeutic approach is mainly symptomatic, as the exact pathogenesis of GT is still unknown. Several etiologic factors have been proposed, including genetic predisposition, stress, hormonal influences, and associations with systemic conditions [2,8,31,33]. Studies have demonstrated a higher prevalence of GT in individuals with psoriasis, suggesting a possible shared pathogenic mechanism. Many observations confirm a link between geographic tongue and psoriasis, showing histological similarity and a common genetic marker: HLA-Cw6 [7,21,22]. Microscopic examination of oral mucosa in GT reveals a thick keratin layer with mixed inflammatory cell infiltrate, thin elongated rete ridges, and epithelial edema with Monro’s abscesses in the keratin and spinous layer. Increased edema, acanthotic epithelium, parakeratotic layers, and a complete absence of filiform papillae are observed in the reddened areas [13]. This strongly corresponds to the histopathologic image of psoriatic lesions. In our study population, histopathological correlations remain speculative due to the small sample (only two patients presented with biopsy results). In a study by Tomb et al. involving 400 psoriasis patients and 1000 controls, the frequency of GT was 7.7% in the study group vs. only 1% in the control group, suggesting a significant association between psoriasis and GT [47]. A significantly higher prevalence of GT in psoriatic patients than in healthy subjects was reported by Picciani et al. [48]. GT was the second most common oral lesion among psoriasis patients in Talaee et al.’s study [49]. Gonzales Alvarez’s et al.’s meta-analysis shows that geographic tongue is three times more frequent in psoriatic patients than in other dermatological patients and the general population [20]. In our latter study on 127 psoriatic patients from Poland, the most common oral lesions were fissured, white-coated (CT), and geographic tongue. A significantly lower prevalence of GT was evident in the group managed with a new class of biological drugs and smokers. There was no association between the oral manifestation and the Psoriasis Area and Severity Index (PASI) score. FT appeared significantly more often in patients who experienced a substantial effect of psoriasis on their quality of life and in smokers [50]. Meanwhile, an association between psoriasis and GT was not confirmed in Chinese observations conducted by Hu et al. [16]. In our study group, we also did not show any association between GT and psoriasis, which was found in only one patient with GT. On one hand, this could indicate some diagnostic biases, including underdiagnosed psoriasis in our study population or insufficiently care being taken when noting patients’ history related to general health conditions. On the other hand, the contradictory results of GT frequency in psoriasis in various studies may result from some regional genetic differences.

Several authors postulated the role of an unbalanced diet and deficiency of specific nutrients, including zinc, iron, and vitamins D and B, in the etiopathogenesis of GT. However, although vitamin D may be helpful in proper keratinization, and its analogs are a recognized treatment for psoriasis, it has been shown that supplementation with large amounts of nutritional supplements does not affect the course of GT [28,31]. This corresponds to the results of our study, where gastrointestinal diseases, very often accompanied by several types of nutritional deficits, were the second most common systemic comorbidity in the study group, found in 24% of subjects. The results of Cigic et al.’s study demonstrated the increased prevalence of celiac disease in patients with geographic tongue. Celiac disease was prevalent among 15% of patients with GT in their study. Moreover, the GT group had a higher concentration of immunoglobulin antibodies in their blood (IgA tTG, IgG tTG, IgA AGA, IGG AGA) [33]. We found celiac disease in 5 of 100 GT patients. Similarly, like in the case of psoriasis, the relatively low incidence of celiac disease in our study compared to the results presented by Cigic et al. may indicate some diagnostic biases related to the underreporting of systemic diseases by the patients. As celiac disease may mimic some other conditions, for example, reflux disease, some patients could also have been underdiagnosed. Gastro-oesophageal reflux disease was the most commonly reported gastrointestinal disease in our study group, reported by 14 subjects. Our latter study found GT in 4 out of 70 patients with Crohn’s disease, and 4% of patients had ulcerative colitis. The prevalence of ulcerative colitis was insignificantly higher in a GT group than in a control group of healthy adults [51]. A study by Zhang et al. also indicated gastrointestinal disorders positively related to GT [52]. An increasing prevalence of GT in infectious gastrointestinal diseases was reported by Katz et al. [53]. Meanwhile, an elevated level of calprotectin in the saliva was observed in patients with GT in a study by Garsjö et al. [54] and in patients with inflammatory bowel disease in a study by Majster [55], which suggests a link between GT and gastrointestinal disorders.

Marks and Tait showed an increased incidence of tissue type HLA-B15 in atopic patients with GT, which supported the theory of a genetic basis of the condition. They observed a positive association between GT and atopy/asthma [25]. GT was also more frequently found in patients with allergies to drugs and foods in a study conducted by Jainkittivong et al. [14]. We found an allergy in 13% and asthma in 8% of our GT population.

The most common oral mucosal disease accompanying GT was candidiasis, found in 13% of the study population. In González-Álvarez et al.’s systematic review, oral candidiasis manifested in 7.6% (24/315) of patients with GT [46]. Contrary to the removable nature of the coating typical of candidiasis, the keratotic lesions of GT cannot be eliminated by scraping and self-treatment, with a tendency to recur within a variable period. The irregular structure of the epithelium in GT may promote infection with several bacterial and fungal species and shifts in the oral microbiota [34]. Thin, depapillated areas are more susceptible to *Candida* infection [9,33,34]. The high rate of *Candida* carriage in patients with geographic and fissured tongue was reported in a study by Dudko et al., who also emphasized that this topic has not been extensively examined so far [24]. Other common oral conditions with GT included recurrent aphthous stomatitis and lichen planus. Fissured tongue and *linea alba* were observed in six persons each. GT’s co-existence with oral lichen planus and recurrent aphthous stomatitis has not been explored extensively in the literature. A similar background of these conditions, involving autoimmune pathways, may explain the higher susceptibility to developing these diseases in one person. There may be some related diagnostic issues, as the overlap of tongue lesions may shield the existence of GT in patients with OLP [52]. However, in Zhang et al.’s study, individuals with RAU (OR = 0.3), B, and OLP (OR = 0.1) had a lower risk of developing GT [52]. GT and fissured tongue were noticed in 60.1% of patients in a study by Jainkittivong et al. [14]. Also, Ghose et al. postulated a genetic linkage between these two conditions [56]. In another study, GT and fissured tongue were associated with chronic granulomatous disease [57]. A study by Zhang et al. proved that FT and gastrointestinal disorders had a significant association with GT [52]. Shulman and Carpenter reported that GT was positively associated with FT among US adults [58]. A total of 34.5% of patients with GT had accompanying FT in Miloglu’s observations [59]. This was in contrast to a cross-sectional study of 600 Malaysian outpatients, which found no significant association between GT and FT [60].

A significantly higher number of patients in this study were non-smokers (87%). When nicotine receptors on macrophages are stimulated, TNF-α production, which is known for its pro-inflammatory action, decreases. That being the case, researchers suggest the possibility of a negative correlation between smoking and the occurrence of GT [32].

The correlation of GT with elevated levels of pro-inflammatory cytokines such as TNF-α and interleukins in GT pathogenesis has been widely discussed [7,22,26]. Single-nucleotide polymorphisms in specific genes can increase susceptibility to GT [22]. A study by Liu et al. revealed that some cases of GT are caused by autosomal dominant IL36RN mutations with incomplete penetrance [26]. Garsjö et al.’s study demonstrated increased calprotectin and interleukin 8 levels in patients’ saliva [54].

According to several reports, some chemotherapeutics (bevacizumab, sorafenib, sunitinib, axitinib) can also lead to GT development [36,38]. Bevacizumab blocks Vascular Endothelial Growth Factor-A (VEGF-A) activity, which impedes the normal reparative capacity of the epithelium and leads to the development of GT lesions [36].

## 5. Limitations of This Study

This study is a retrospective observational survey and, as such, has many limitations. These include the study’s ununiform diagnostic approach, where the doctors were not calibrated. An uncalibrated diagnostic approach among clinicians could lead to variability in GT diagnosis and may interfere with the precision of the results. The lack of a control group and missing follow-up data preclude a more profound comparative analysis. The sample size was relatively small, and the participants were recruited retrospectively, including a limited 10-year period. Selection bias may exist, since patients originated from a single outpatient clinic, so they may not represent the general population. The histopathological data were insufficient to state any conclusions. Additionally, a regression analysis was not conducted, and there was limited control over the nature and quality of the predictor variables. Nevertheless, this analysis is valuable, as it sheds additional light on the epidemiological characteristics of GT patients from Northeastern Europe.

## 6. Conclusions

The results of this study reveal that GT affects patients of both genders at a similar frequency. The mean age of patients was 51.6 years, mostly non-smokers of an urban origin. Subjective complaints often followed GT. Given the high association rates, GT screening should include cardiovascular and gastrointestinal evaluation.

As the etiology of GT remains elusive, further research is required. Due to the chronic course of the condition and its unpredictable mode of recurrence, dentists should provide detailed information to GT patients and suggest possible therapeutic approaches to reduce their symptoms. Patient counseling on GT’s benign but symptomatic nature may reduce anxiety.

## Figures and Tables

**Table 1:** Comparison of classifications of geographic tongue and geographic stomatitis, according to Hume and Netto [1,6].

Hume’s Classification	
1	Geographic tongue, without geographic lesions elsewhere in the mouth
2	Geographic tongue, accompanied by geographic lesions elsewhere in the mouth
3	Atypical, fixed, or abortive, tongue lesions, whether or not accompanied by geographic lesions elsewhere in the mouth
4	Geographic lesions elsewhere in the mouth without the presence of a geographic tongue
Netto et al.’s classification	
1	Geographic tongue
2	Geographic stomatitis without tongue involvement
3	Geographic stomatitis with geographic tongue
4	Geographic tongue associated with cutaneous diseases (i.e., psoriasis)
5	Geographic stomatitis without tongue involvement associated with cutaneous diseases (i.e., psoriasis)
6	Geographic stomatitis with geographic tongue associated with cutaneous diseases (i.e., psoriasis)

**Table 2:** Demographic characteristics of the study group, considering gender distribution, residence, and smoking.

	n (%)	*p*
Total	100 (100%)	
Gender distribution		
Females	52 (52%)	0.5716
Males	48 (48%)	
Residence		
Rural	19 (19%)	<0.0001
Urban	81 (81%)	
Habits		
Smokers	13 (13%)	<0.0001
Non-smokers	87 (87%)	

**Table 3:** Age of study participants in relation to gender.

	Total	Females	Males	*p*
Age (mean ± SD, range [years])	51.6 ± 22.2 16–101	57.0 ± 20.8 16–101	46.0 ± 22.5 18–95	0.0126

**Table 4:** Symptoms in study participants with GT, stratified by gender and age group.

	n, % of TSP *	F (n = 52)	M (n = 48)	*p*	<40 y.o. (n = 43)	>40 y.o. (n = 57)	*p*
Number of patients reporting complaints	85 (85%)	46	39	0.3167	34	51	0.0150
Number of patients not reporting any complaints	15 (15%)	6	9	0.4206	9	6	0.4206
Total number of complaints	139	82	57	0.0029	52	87	<0.0001
Symptom							
Burning sensation	57 (57%)	36	21	0.0188	22	35	0.0417
Pain	39 (39%)	21	18	0.5924	17	22	0.3722
Xerostomia	22 (22%)	12	10	0.6513	5	17	0.0067
Bleeding	4 (4%)	1	3	0.3124	2	2	1.0000
Taste disturbances	3 (3%)	3	0	0.0810	1	2	0.5607
Paresthesia	2 (2%)	1	1	1.0000	1	1	1.0000
Excessive saliva production	2 (2%)	1	1	1.0000	1	1	1.0000
Other	10 (10%)	7	3	0.1944	3	7	0.1944

* TSP—total study population (100). F—females. M—males. y.o.—years old.

**Table 5:** Histopathological findings concerning patients’ age, sex, comorbidities, and habits.

Patient	Age	Sex	Systemic Comorbidities	Oral Comorbidities	Habits	Histopathologic Findings
RR	89	F	HT	Denture-induced ulcer	NR	Inflammatory infiltrate without dysplasia
TJ	71	M	COPD, HT, cardiovascular disorders, prostatic hyperplasia	Lichen planus, oral melanotic macule	NR	Hyperkeratosis and lymphocytic infiltrate

RR, TJ—patients’ initials. F—females. M—males. COPD—chronic obstructive pulmonary disease. HT—hypertension. NR—not reported.

**Table 6:** Oral diseases and lesions other than GT found in the study group.

Oral Condition	n = % of TSP *
Candidiasis	13
Recurrent aphthous stomatitis (RAS)	10
Fissured tongue (FT)	6
*Linea alba*	6
Lichen planus (LP)	5
Denture stomatitis	3
Trauma-induced ulcer	3
Gingivitis	3
Angioma	3
Cheilitis	2

* TSP—total study population (100).

**Table 7:** Systemic diseases in the study group.

Systemic Diseases	n (%)	F (n = 52)	M (n = 48)	*p*	<40 y.o. (n = 43)	>40 y.o. (n = 57)	*p*
Disease reported	76 (76%)	43	33	0.1452	24	52	<0.0001
Disease not reported	24 (24%)	9	15	0.1917	19	5	0.0023
Cardiovascular diseases	37 (37%)	20	17	0.5849	3	17	0.0047
Gastrointestinal diseases	24 (24%)	16	8	0.0817	7	17	0.0296
Thyroid disorders	18 (18%)	17	1	0.0001	8	10	0.6212
Type 2 diabetes	15 (14%)	7	8	0.7883	1	6	0.0544
Allergy	13 (13%)	6	7	0.7742	6	7	0.7742
Asthma	8 (8%)	3	5	0.4705	3	5	0.4705
Neurological diseases	3 (3%)	2	1	0.5607	1	2	0.5607
Psychiatric disorders	3 (3%)	1	2	0.5607	0	3	0.0810
Psoriasis	1 (1%)	1	0	0.3161	0	1	0.3161

## Data Availability

All data underlying the results are available from the corresponding author upon request.
